# Durability of MgO-based binders: a critical review from RILEM TC 311-MBC

**DOI:** 10.1617/s11527-026-03076-7

**Published:** 2026-04-20

**Authors:** Shishir Mundra, Vincent Hallet, Ellina Bernard, Nirupama Kamala Ilango, Hoang Nguyen, Fabio E. Furcas, Navid Ranjbar, Dan Meng, Juan Pablo Gevauden, Matthias Mueller, Timothy Aiken, Barbara Lothenbach

**Affiliations:** 1 Institute of Building Materials, ETH Zürich, Laura-Hezner-Weg 7, 8093 Zurich, Switzerland; 2 Swiss Federal Laboratories for Materials Science and Technology (EMPA), Laboratory for Concrete and Asphalt, 8600 Dübendorf, Switzerland; 3 Fibre and Particle Engineering Research Unit, University of Oulu, Pentti Kaiteran Katu 1, 90014 Oulu, Finland; 4 Department of Mechanical Engineering, Technical University of Denmark, 2800 Kgs. Lyngby, Denmark; 5 School of Civil and Environmental Engineering, Nanyang Technological University, 50 Nanyang Avenue, 639798 Singapore, Singapore; 6 Department of Architectural Engineering, Department of Materials Science and Engineering, The Pennsylvania State University, University Park, PA 16802 USA; 7 F. A. Finger-Institut Für Baustoffkunde, Bauhaus-Universität Weimar, Fakultät Bau- Und Umweltingenieurwesen, Weimar, Germany; 8 Translink, 22 Great Victoria Street, Belfast, Co Antrim, BT2 7LX UK

**Keywords:** Magnesia-based cements, Durability, Magnesium oxychloride cements, Magnesium oxysulfate cements, Magnesium carbonate cements, Magnesium silicate cements

## Abstract

The urgent need to decarbonise the cement industry has motivated global research on MgO-based cements, including magnesium oxychloride (MOC), oxysulfate (MOS), carbonate (MC), and silicate (MS) cements. While this new class of cements may offer material advantages over Portland cement, questions surrounding their long-term durability continue to limit their broader implementation. This critical review, prepared for RILEM TC 311-MBC, evaluates the durability literature on these four types of MgO-based binders. Findings show that while MOC and MOS cements exhibit attractive early-age strength, they suffer rapid strength loss under sustained exposure to moisture because their main hydrates are highly soluble. Field test data is critically needed. MC binders, dominated by hydrous carbonate-bearing brucite, exhibit good resistance to chloride and sulfate environments; however, their strength decreases with increasing humidity, and accelerated carbonation can trigger expansion or micro-cracking, depending on the availability of pore spaces. Contrastingly, MS cements develop dense M-S–H matrices with permeability and frost performance on par with, or better than, PC-based concretes. This promising class of cements resists natural carbonation but remains sensitive to micro-crack formation when nesquehonite precipitates in high CO_2_ environments. Across all MgO-based binders, reinforcement corrosion is driven by moisture transport rather than by differences in passivation due to the near-neutral pH of their pore solutions. The research community is encouraged to close these gaps so to advance the formulation of durable and environmentally sustainable MgO-based cements.

## Introduction

Magnesia (MgO)-based binders represent a class of cementitious materials that hold significant promise as an alternative to traditional Portland cement (PC). MgO-based binders have a long history, with their use as a construction material dating back over a century. Historically, MgO-based cements were valued for leveraging abundant local resources and offering perceived commercial benefits over PC, such as enhanced aesthetic or mechanical properties [1]. In recent decades though, the primary motivation to develop and adopt MgO-based binders has been rooted in environmental considerations. However, until now their usage has been limited to niche applications, and very little magnesium is used for cement production. Currently, MgO used in cementitious materials is primarily sourced from calcined magnesite (MgCO_3_). Extraction of reactive MgO may require lower calcination temperatures when compared to extraction of CaO from CaCO_3_. However, extraction of reactive MgO from MgCO_3_ poses similar environmental concerns like PC and may even result in overall higher process emissions when compared to PC [2]. To mitigate the emissions related to the decarbonation of the precursor, the use of alternative feedstocks, such as MgO from desalination brine/seawater or from magnesium silicate minerals (e.g. olivine, (Mg,Fe)_2_SiO_4_) is an active area of research, as discussed in detail in [3].

MgO-based binders represent a diverse range of cementitious materials, such as magnesium oxychloride (MOC)/oxysulfate (MOS) cements, magnesium phosphate cements (MP), magnesium carbonate cement (MC) and magnesium silicate cements (MS). Each of these binders vary in their chemistry and are significantly different from Portland cement and other modern binders such as alkali-activated materials. The hydrates formed from MgO-based cements differ widely. MOC and MOS cements form different metastable and stable hydrous oxysalt phases, as discussed in recent reviews [1, 4]. MP cements harden by the reaction of soluble acid phosphates such as NH_4_H_2_PO_4_ or KH_2_PO_4_ with MgO, resulting in the formation of mainly struvite-like phases: NH_4_MgPO_4_⋅6H_2_ or KMgPO_4_⋅6H_2_O [5, 6]. MS cements harden due to the formation of magnesium silicate hydrate (M-S–H) phases from the reaction of MgO and silicate in the presence of water [1, 7]. Under ambient conditions, the hydration of MgO with carbonates leads to the formation of various metastable hydrated magnesium carbonate phases (MC cements) [3, 8]. One of the main pathways presently being investigated is based on a blend of MgO and reactive magnesium carbonates such as hydromagnesite or nesquehonite, leading to the formation of low-crystalline brucite containing carbonate and water, exhibiting good compressive strength [9, 10].

MOC (also known as “Sorel cements”), MOS and MP cements find use in niche applications. MOC cements are used in applications such as temporary oil-well cements with acid-soluble additives for easy removal when needed, sealing brine intrusions in rock salt and potash mining, and stabilizing nuclear waste repositories in salt mines. MOS cements find use as lightweight construction materials, in insulating boards and fire-resistant panels. MP cements find application in rapid patch repair work and can potentially be used as a nuclear waste immobilisation matrix. MgO-based cements containing silica and/or carbonates have low pH values, can exhibit high strength and their production is potentially associated with low CO_2_ emissions [11] when the MgO source is not MgCO_3_ as discussed in [3], enabling their use for a diverse range of applications and serving as an alternate to Portland cements for various products.

Although these binders present an opportunity to develop more environmentally friendly cementitious materials, one of the major hurdles lies in the lack of data concerning their long-term durability. In this manuscript, as part of RILEM TC 311-MBC, we aim to provide a critical review of the available literature on the durability of several MgO-based binders, namely, MOC, MOS, MC, and MS cements. The primary durability concerns addressed in this review include corrosion of the steel reinforcement, carbonation of the concrete cover, sulfate attack, frost attack, and water permeability. This review aims to provide a comprehensive overview of the durability concerns associated with these binders, laying the groundwork for future studies and practical applications.

## Durability indicators

Defining appropriate durability indicators is crucial for assessing the long-term performance of cementitious materials, including emerging MgO-based binders. Among these, leaching refers to the dissolution and transport of soluble phases from the hardened matrix, often accelerated under aggressive environmental conditions, which can compromise structural integrity and alter the composition of the pore solution within the cementitious matrix. Water permeability, on the other hand, provides insight into the material’s pore structure and resistance to water transport, serving as a primary indicator of how easily aggressive agents can penetrate the concrete. In moderate and cold climates, frost action can damage saturated concrete. Especially in combination with de-icing salt intensive surface damage can occur. Novel binders typically have an inferior frost and salt frost resistance than PC, hindering the implementation of such binders in frost-prone environments. Closely related, carbonation involves the reaction of CO_2_ with alkaline components, leading to a decrease in the pH of the pore solution that can result in the depassivation of the steel reinforcement. For MgO-based binders, the carbonation mechanism and products can differ significantly from PC, necessitating a thorough understanding. Sulfate attack represents another major degradation mechanism, where external sulfates react with binder components, potentially leading to expansive products and cracking. Other critical durability indicators include chloride ingress, which is especially relevant in marine or de-icing salt environments. The ingress of chloride ions can break down the passive film on reinforcement steel, initiating corrosion. The corrosion of the steel reinforcement is a key indicator of potential premature structural degradation. Assessing the corrosion resistance in MgO-based binders requires a comprehensive understanding of the solid phase assemblage, pore structure and water permeability, pore solution chemistry and pH of the binder matrix. In this review, we critically assess hydrated MgO-based binders with respect to various durability indicators. Accordingly, the terms ‘cements’ and ‘binders’ used hereafter specifically refer to the hydrated forms of MgO-based binders.

### Leaching

Both magnesium oxychloride (MOC) and oxysulfate (MOS) cements show very little stability when exposed to high moisture environments. When exposed to water, the main binding phases in MOC cements, 3Mg(OH)_2_·MgCl_2_·8H_2_O and 5Mg(OH)_2_·MgCl_2_·8H_2_O, dissolve, resulting in loss of strength of the cement. Upon 28 days of immersion in water, Deng [12] observed an average decrease of 37% in the compressive strength of MOC mortars. In high ionic strength brines bearing Mg^2+^ and Cl^−^, the leaching of Mg^2+^ and Cl^−^ from hydrate phases has been observed to be much lower compared to leaching experiments in water [13]. Stewart [14] and Deng [12] suggested the addition of small quantities of phosphates to improve the water resistance of MOC cements. It has been proposed that phosphates can decrease the concentrations of Mg^2+^ in solution and favour the formation of 5Mg(OH)_2_·MgCl_2_·8H_2_O. However, the precise mechanism by which phosphates influence the water resistance of MOC cements is unknown and requires further research [12, 14]. Additionally, to decrease the dissolution of Mg^2+^, partial replacement of MgO in MOC cements with supplementary cementitious materials such as metakaolin has also been suggested [15].

MOS cements show poor leaching resistance similar to MOC cements, even though the solubility of the sulfate bearing 5-phase hydrate (5Mg(OH)_2_·MgSO_4_·7H_2_O) phase is lower than the hydrate phases formed in MOC cements. Unreacted MgO can react with water at later stages to form additional porous Mg(OH)_2_, associated with a volume expansion of ~ 147%, resulting in the formation of microcracks that reduce strength and increase water permeability. Some authors have studied the use of acids/citrate/hydromagnesite to inhibit further hydration of MgO [16, 17], however, even after 180 days residual MgO reacts with water to form Mg(OH)_2_.

MC cements based on blending MgO with reactive magnesium carbonates (hydromagnesite) exhibit significant swelling as well as a strength reduction when cured submerged in water [18, 19]. In fact, a gradual decrease in strength is observed with curing at increasing relative humidities. However, the low-crystalline hydrous carbonate containing brucite (HCB) that makes up the binder in these systems does not dissolve upon submersion in H_2_O and thus cannot explain the reduction in strength. Other mechanisms, such as the formation of other phases, phase changes or microcracking due to swelling were investigated but did not yield a satisfactory explanation for the observed strength reduction. Zhu et al. [19] hypothesised that the decrease in mechanical properties upon moisture uptake is due to the intrinsic impact of adsorbed water on the microstructure. The cause of the strength reduction should be further investigated to aid in the development of mitigation strategies.

Hydrated MS cements show excellent water resistance, as samples cured under water retain high strength [20]. The presence of sufficient water allows the initially formed brucite to further convert into M-S–H resulting in a 28-day compressive strength > 45 MPa. With prolonged curing, Mg(OH)_2_ content of the cement decreases gradually, forming additional M-S–H, which is the main binding component in these types of cements. Although a high water resistance has been observed, systematic evaluations of leaching characteristics of MS cements are still missing to confirm their resistance in structural applications.

### Permeability

The high solubility of the hydrates in MOC cements results in their dissociation, which significantly results in high water permeability. The addition of silica fume, aluminosilicates, and phosphate salts, has been shown to improve water resistance and decrease the permeability to 0.20–2.45 × 10^−4^ cm/s [21]. MOS cements also show high water permeability due to soluble hydration products, and additionally, their long-term performance under moisture exposure remains uncertain [22]. It is worth highlighting that in addition to water resistance (i.e., direct exposure to water), MOC and MOS are also susceptible to phase changes when exposed to moisture in the air (i.e., high levels of humidity) [22]. Their use in conditions with high humidity or with exposure to water must be avoided.

Limited data on the gas or water permeability of mortars or concrete made with MC binders are available in the open literature. Meng et al. [23] performed water permeability test on pervious concrete samples with reactive MgO. Different void ratios in the concrete were achieved by varying the binder-to-aggregate ratio. The water permeability coefficient of the samples cured at 10% CO_2_ (30 ± 1.5 °C and 80 ± 5% RH) for 7 days ranged from 0.01 to 0.33 cm/s. As expected, a higher void ratio in the specimen resulted in a higher permeability coefficient. With an increase in CO_2_ curing time, a reduction in the permeability coefficient (from 0.33 cm/s at 7 d to 0.23 cm/s at 28 d) was observed particularly for samples with higher porosity. This was due to the formation of a dense microstructure from the continuous carbonation of MgO, which led to the precipitation of hydrated magnesium carbonates (HMCs). Similarly, curing porous reactive MgO-based blocks at 20% CO_2_ concentration (20 °C and 70% RH) led to a reduction in water permeability by 50–90% compared to porous blocks cured at ambient atmospheric conditions [24]. The decrease in permeability was attributed to the reduction in pore space due to the formation of HMCs.

MS cements show comparable or increased permeability compared to PC. Tran et al. [25] observed that the oxygen permeability of M-S–H (magnesium silicate hydrate) binders is comparable to that of hydrated Portland cement (PC) after 7 days of curing. However, as hydration progresses, the permeability coefficient of PC decreases, while that of M-S–H binders increases. The increased oxygen permeability of M-S–H binders was explained by (1) an increase in pore connectivity and (2) microcracking associated with M-S–H formation. Dhakal et al. [26] conducted oxygen permeability tests on M-S–H concrete specimens made from MgO and metakaolin. They found that the permeability of M-S-H concrete was lower than that of PC concrete, attributing this improvement to the pore-filling effect of hydrotalcite phases formed within the M-S–H system. The limited amount of experimental data indicates a need for further systematic investigations of the long-term changes in permeability.

### Frost attack

Frost attack occurs when freeze–thaw cycles coincide with moisture saturation in concrete. The situation exacerbates when additional salts, whether from de-icing salts or seawater, interact with the concrete, leading to what is known as salt frost attack. As MOC and MOS cements are prone to moisture damage, they are typically not used as structural material [1, 27], which precludes their use in concretes that are exposed to frost attack. As a result, there is no published research on frost attack specifically related to these types of cement in structural applications, but only for non-structural building materials (e.g. [28, 29]). Accounting for the susceptibility against moisture it can be concluded that concrete based on MOC and MOS should have an inferior frost resistance when compared to Portland cement-based materials.

Only scarce research has been conducted on the topic of frost or salt frost scaling resistance of concretes with MC or MS cements. In a study on the performance of binders based on MgO and activated serpentine (1:1) with additions of MgCl_2_, Lee et al. [30] conducted freeze–thaw resistance tests according to Korean standard KS F 2456. In this test procedure frost resistance is evaluated by comparing the compressive strength of concrete after 100 freeze–thaw cycles with that of concrete without frost exposure. With 20% MgCl_2_ a good frost resistance was achieved, with 95% residual compressive strength. For 10% MgCl_2_ only 80.5% residual compressive strength was observed after the freeze–thaw test. The improved performance for 20% MgCl_2_ can be attributed to the accelerating effect of MgCl_2_, which led to a notably higher initial compressive strength and therefore probably to a more refined pore structure. Though no comparison to PC concrete is given here, these results show that MS based concrete can have a good frost resistance, which also agrees with the relatively low permeability observed in MS cements (see Sect. 2.2).

In PC based concretes for frost and especially salt frost exposed structures and elements, artificial air entrainment is used to ensure good durability. Ruan and Unluer [31] observed that air entrainment can be easily achieved with MgO-based binders. Therefore, an important prerequisite to produce frost- and salt frost resistant concrete with magnesia-based binders is fulfilled.

### Carbonation

MOC and MOS cements are typically not used as structural material [1, 27], and therefore carbonation of such cements is little studied. However, it has been observed that natural carbonation of magnesium oxychloride cements containing brucite resulted in the formation of magnesium carbonates such as hydromagnesite (Mg_5_(CO_3_)_4_(OH)_2_·4H_2_O) and chlorartinite (Mg_2_(CO_3_)Cl(OH)·2.5H_2_O), while the volume of brucite and oxychlorides decreased [32].

The main hydrates formed in MOC cements have been observed to react slowly with CO_2_ in the atmosphere to form nesquehonite (MgCO_3_·3H_2_O), hydromagnesite (Mg_5_(CO_3_)_4_(OH)_2_·4H_2_O), or chlorartinite (Mg_2_(CO_3_)Cl(OH)·2.5H_2_O) [32–34]. The amount of bound CO_2_ increases with age as well as with CO_2_ concentration [32]. Accelerated carbonation (20% CO_2_, 70% RH, 20 °C) of MOS cements over 28 days resulted in a gradual decrease in pH from 10.1 to 9.9 within the first 5 mm of the mortar surface [35]. On their specimens, Ba et al. [35] observed MgCO_3_ and a refinement of the pore structure. However, it is difficult to conclude whether MgCO_3_ formed as a result of carbonation or pre-existed in the paste precursor.

Mg-carbonates such as hydromagnesite and magnesite have their natural deposits on Earth’s surfaces [36]. Magnesite (MgCO_3_) is the thermodynamically most stable magnesium carbonate [37, 38]. Although magnesite has been attracting great interest for the permanent storage of CO_2_, its formation remains an enigma. Magnesite requires very high temperatures and pressures to be synthesized under laboratory conditions [39], but frequently occurs in natural sedimentary minerals at Earth’s (sub) surface. The phenomenon links to a complex relationship between a strong solvation shell of Mg^2+^ in aqueous media [40, 41] and the lattice limitation on the spatial configuration of CO_3_^2−^ groups in magnesite [42]. This also partially explains the absence of magnesite after the carbonation of Mg-rich phases at ambient temperature and pressure. The carbonation of MgO at ambient conditions can be slow, taking several years similar to natural weathering processes [43]. The most relevant information on the long-term carbonation of MgO at ambient conditions available is based on the land-spreading concept developed with an aim to sequestrate atmospheric CO_2_. In this context, McQueen et al. [44] predicted to achieve about 90% carbonation of MgO to form magnesite within a year when exposed to atmospheric CO_2_. However, experimentally, Rausis et al. [45] found that only 3–18 wt% of MgO (would react with atmospheric CO_2_ to form dypingite [Mg_5_(CO_3_)_4_(OH)_2_· ~ 5H_2_O] in 1 year when subjected to wetting and drying cycles to simulate weathering. Here, the limiting factor was the rate of CO_2_ ingress, which was influenced by water content, porosity, and permeability [45].

The CO_2_ concentration and extent of diffusion significantly influence the rate and degree of carbonation of MgO [47]. Dung et al. [47] observed an increase in carbonation rate with increase in CO_2_ concentration from 5 to 20%, however, both samples exhibited similar 28-day strength. The rapid formation of HMC in the case of 20% CO_2_ at the surface results in a dense microstructure and limits further diffusion of CO_2_. Therefore, MC cement when exposed to suitable conditions with a high rate of CO_2_ supply, may be susceptible to a high degree of carbonation.

**Fig. 1:**
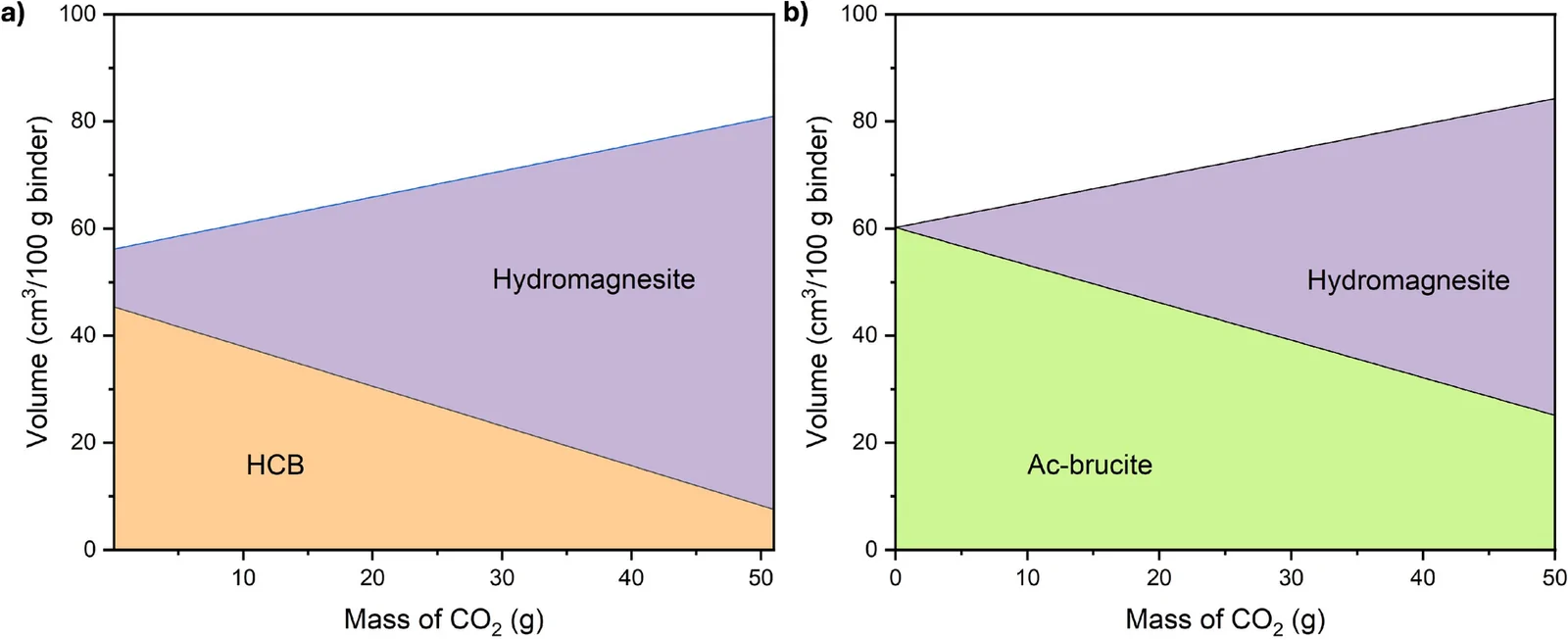
Thermodynamic modeling of phase evolution during carbonation (1% CO_2_) of **a** 70%–30% MgO/hydromagnesite blended cements and **b** MgO cements with the addition of acetate. The pH is calculated to remain constant at 9.9 and 9.1 during the carbonation of HCB and Ac-brucite, respectively. The x-axis represents the stepwise addition of CO_2_ in grams (g), mimicking the contact of the binder to air containing 1% v/v CO_2_. HCB, hydrous carbonate-containing brucite; Ac-brucite, acetate-containing brucite

Further, unreacted Mg(OH)_2_ in MgO-based binders [8] and hydrous carbonate-containing brucite (HCB) [48] can be susceptible to carbonation during service-life. Accelerated carbonation of HCB formed in MgO/hydromagnesite blends [48] and of reactive MgO binders with acetate [49] is modelled thermodynamically (Fig. 1) using the thermodynamic dataset as compiled in [48] and [50] (suppressing the potential magnesite formation). Hydromagnesite is predicted to be the stable carbonation product in both systems. It should be noted that the growth rate of hydromagnesite depends on temperature, and its growth is much slower at ambient conditions than at elevated temperatures [51]. Hydromagnesite was found to precipitate at 50 and 80 °C within 3 months, but only after 1 year at 20 °C [52].

**Fig. 2:**
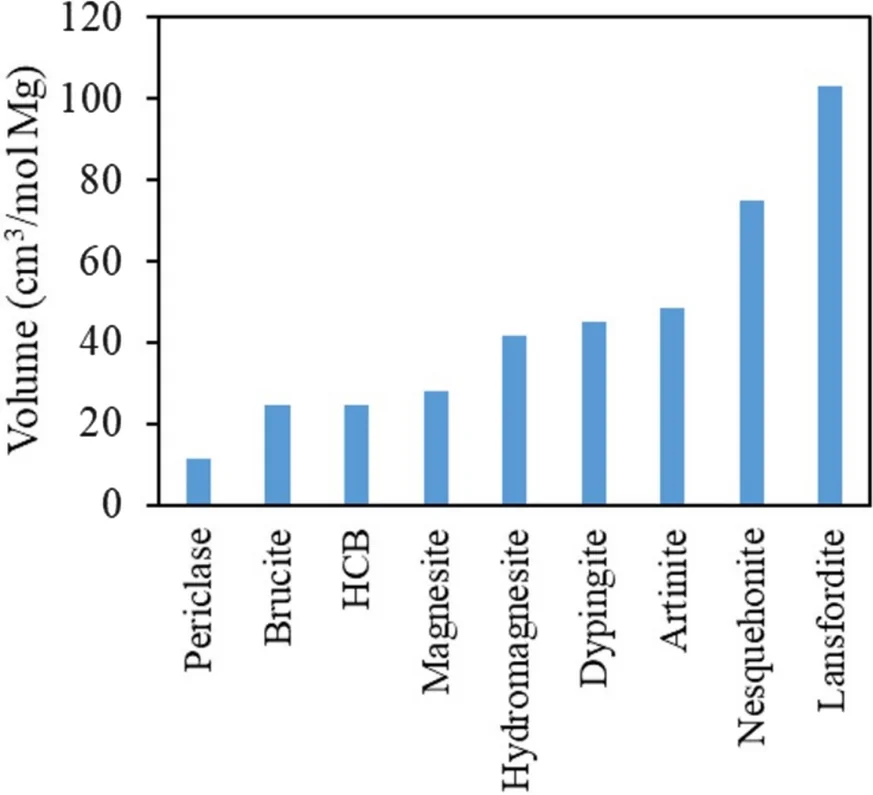
Volume of different magnesium phases per mole of Mg atoms at 25 °C, 1 bar [1, 8]

Thermodynamic modelling (Fig. 1) indicates that carbonation of HCB or Ac-brucite [46] leads to the formation of hydromagnesite, accompanied by an increase in solid volume, which could result in structural instabilities and or an increased strength. There is a significant volume difference between MgO and its hydration and carbonation products. For example, the molar volume of MgO is 11 c^3^/mol-Mg, while that of brucite and HCB is more than twice (25 cm^3^/mol-Mg). At the same time, crystalline hydrated magnesium carbonates have volumes ranging from 42 to103 cm^3^/mol-Mg, respectively (Fig. 2, [1, 8]).

The transition from one phase to another is a function of environmental conditions and time [37]. Depending on the nature and rate of the phase transition and the initial pore structure, the formation of Mg-carbonate cement can lead to volume instability and microcracking [23, 53] or, conversely, microstructural densification [54]. A study comparing 56 days of air curing, water curing, and water–air cycles (7 days of water and 7 days of air) of light-burned MgO-based mortar observed shrinkage of 0–120 µm/m, and expansion of 500–960 µm/m and 460–770 µm/m, respectively [55]. This is due to the formation of more brucite in the mortar exposed to water curing and water–air cycle conditions. No crystalline carbonate phases were detected in the samples cured under these two conditions according to X-ray diffraction. However, the presence of poorly crystalline or amorphous carbonate phases was evidenced by phenolphthalein spray and FTIR results. Under accelerated carbonation (20% CO_2_, 30 °C and 80% relative humidity), the compressive strength of carbonated MgO paste (w/c 0.55) was found to be 11% lower at 28 days than at 14 days, which was attributed to excessive carbonation leading to internal stress and cracking [47] due to the formation of nesquehonite and hydromagnesite [53]. A recent study [23] showed that MgO-based pervious concrete with a low void ratio (0.15) developed microcracks after 7 days of accelerated CO_2_ curing and then 11 water-CO_2_ cycles. In contrast, specimens with relatively high void ratios (0.2–0.3) did not exhibit obvious microcracks due to larger space available for expansion [23].

M-S–H formation has been observed in cements and concrete (Portland cement [56–60] and Roman concrete [61]) under long-term exposure to Mg-enriched environments. This is probably due to the higher stability of M-S–H as the Mg-Si bond is [56] at ambient conditions more favorable than that with carbonates [62]. M-S–H also forms during the production of reactive silicates and Mg-carbonate phases from the magnesia silicate minerals such as olivine or serpentine [63]. Understanding the response of M-S–H towards carbonation is crucial to enable its use in construction.

**Fig. 3:**
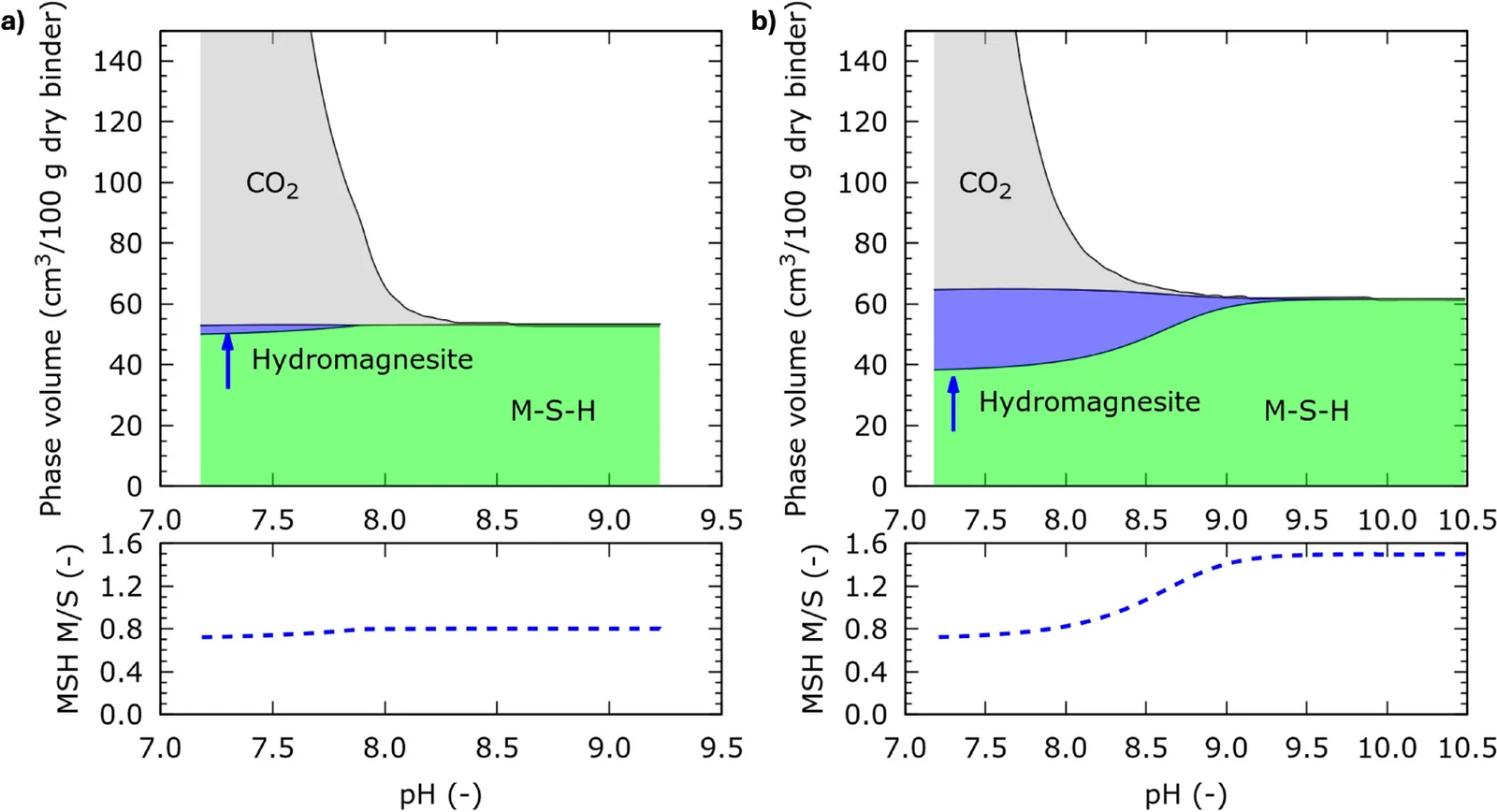
Thermodynamic modelling of the change in phase volume for MS cements (with Mg/Si molar ratio of **a** 0.8 and **b** 1.5) with a water/binder = 1 when exposed to pure CO_2_. The amount of CO_2_ added increases from right to left

To date, only a few studies have investigated the carbonation of MS cements and the main binding M-S–H phase. Aqueous carbonation at ambient temperature of suspensions of 8-year-old M-S–H showed that M-S–H with Mg/Si = 0.8 resists carbonation very well and M-S–H remained stable even towards neutral pH [64]. However, some Mg was released into the solution, and some amorphous silica formed. M-S–H’s with higher Mg/Si ratios (1.1 and 1.5) released Mg^2+^ to attain a stable M-S–H phase with Mg/Si = 0.7. In a less diluted system, such as in the case of M-S–H pastes, nesquehonite could be expected to form [65], in addition to M-S–H or hydromagnesite after longer reaction times. This is illustrated through thermodynamic modelling for a system with a high water/binder ratio (w/b = 1) in Fig. 3, similar to the thermodynamic simulations presented in [64]. The calculations indicate that prolonged carbonation and elevated CO_2_ concentration lowers the pH values to near neutral pH and the formation of traces of hydromagnesite can be expected, even for M-S–H with low Mg/Si = 0.8 (Fig. 3a). Higher Mg/Si molar ratio mainly increases the amount of hydromagnesite that is formed upon carbonation (Fig. 3b). Experimentally, nesquehonite is often observed to form first that eventually to transform into the more stable hydromagnesite [64].

Dry carbonation (~ 20 bar CO_2_ at room temperature) of M-S–H and M-A-S-H powders with resulted in slight rearrangement to M-S–H and the release of silica in the case of low Mg/Si (Mg/Si = 0.8) M-S–H [64]. M-S–H and M-A-S–H with higher Mg/Si carbonated with the formation of nesquehonite. Steam carbonation (18 bar CO_2_ and 200 °C) [64] did not generally modify M-S–H and M-A-S–H powders (except reducing the Mg/Si), however, magnesite was reported to form in the case of M-S–H with a high Mg/Si ratio. Similarly, accelerated carbonation (20% CO_2_) of 1-month aged M-S–H pastes with Mg/Si ~ 1 for 14 days resulted in the precipitation of some nesquehonite, along with a reduction in porosity and an increase in compressive strength compared to the non-carbonated samples [65]. However, extended durations of accelerated carbonation resulted in the development of microcracks due to the formation of nesquehonite, leading to lower compressive strength. It appears that elevated temperatures and/or pressures are required to carbonate M-S–H with high Mg/Si ratios and to form Mg-carbonates. Although at high-CO_2_ pressure magnesium carbonate forms, the resistance of M-S–H towards natural carbonation is very high and presents promising prospects for future applications of these cements.

### Chloride ingress

Chloride ingress is of little interest for MOC and MOS cements, as they are typically not used as structural material [1, 27]. MOC cements exhibit a high chloride concentration within the concrete and at the steel–concrete interface, which will lead to corrosion [66, 67].

**Fig. 4:**
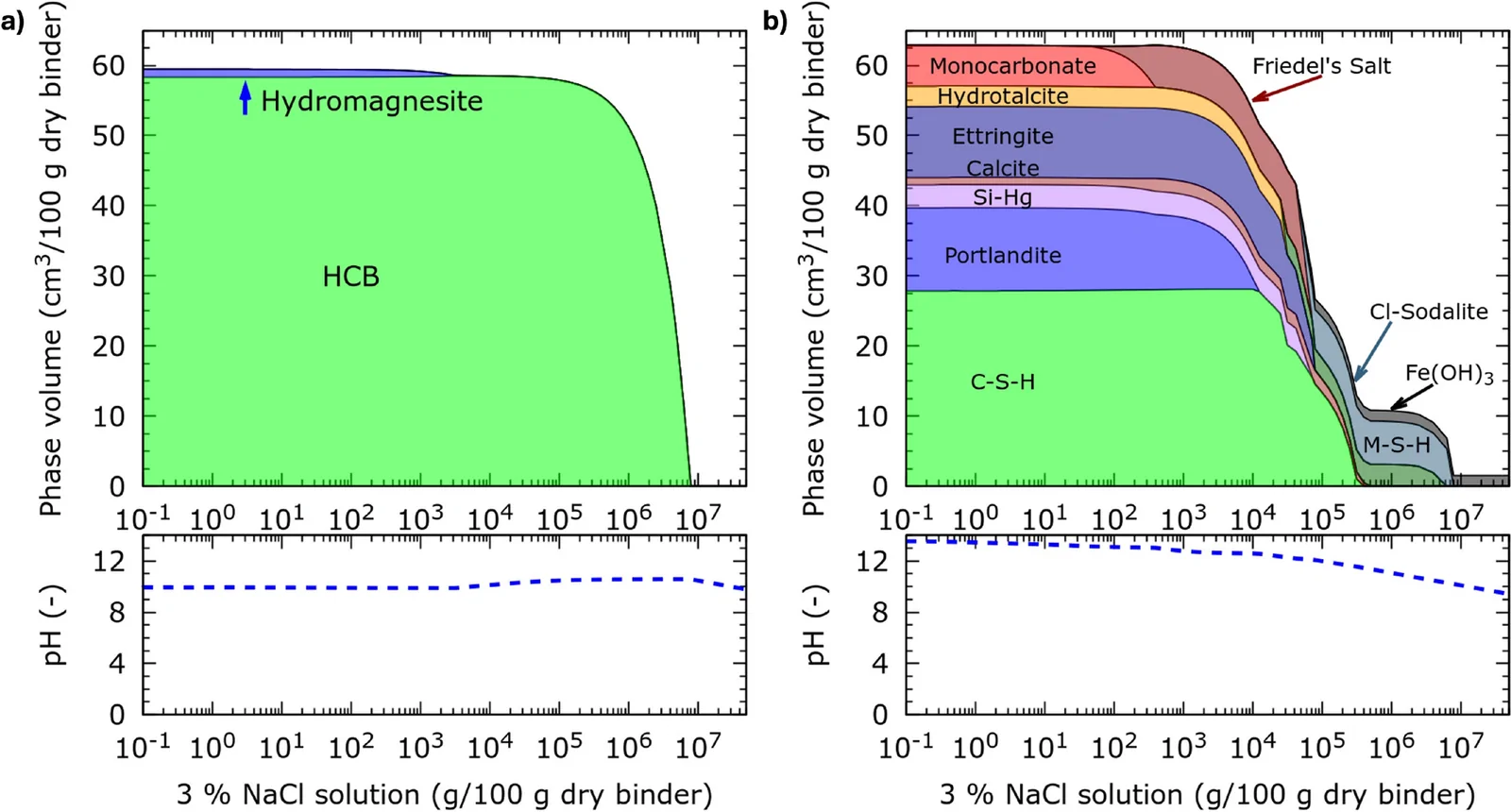
Modelled phase assemblage and associated volume changes upon contact of **a** MgO/hydromagnesite (90/10 mass ratio, water/binder mass ratio = 1) and **b** PC (water/binder mass ratio = 0.4) with 3% NaCl solution. Thermodynamic data on 3-1-8 and 5-1-8 MOC phases from [71, 72] and magnesium carbonates from [48]. PC modelling as described in [73] using the PC composition from [74]

A recent study [68] has revealed that HCB mortars (w/b = 0.5) based on MgO/hydromagnesite feature satisfactory chloride resistance for the exposure classes XD2 (CH) and XD3 (CH), in line with the recommendations of the Swiss Federal Roads Office (FEDRO) [69]. The rapid chloride ingress test, as performed according to the Swiss standard SIA 262/1 Appendix B [70], yields chloride migration coefficients of D_Cl_ = 0.6 ± 0.3 × 10^−12^ m^2^/s and of D_Cl_ = 1 ± 0.6 × 10^−12^ m^2^/s for mortars made from 90/10 and 70/30 wt% MgO/hydromagnesite, respectively [68]. It should be noted that in this standard, the exposure solutions have a substantially higher pH of 13 than the expected pH of 10.5 in the pore solution of these binders [48]. An investigation into the chloride resistance using lower pH solutions is warranted to confirm satisfactory chloride resistance. Upon submersion of those hydrated MgO/hydromagnesite paste into 0.2 M KOH + 3% NaCl solutions, no magnesium (oxy)chloride phases were observed within 28 days [18]. No clear changes were visible in the reflexes of the HCB phase in the X-ray diffraction pattern upon immersion for 28 days in NaCl + KOH solution [68]. In the presence of chlorides, the thermal degradation peak of HCB in TGA shifted to slightly lower temperatures, which may possibly indicate an impact of the chlorides on the HCB. These observations are in line with expectations from thermodynamic modelling (Fig. 4a), where no phase changes are expected in 3% NaCl with HCB remaining stable. Only at very high water to solid contents, > 10^6^ g solution/100 g binder, leaching is expected (Fig. 4a). In contrast, the modelling results in Fig. 4b show that PC leaches much more and tends to decompose in significantly lower amounts of NaCl solution (10^4^ g solution/100 g binder).

Little is known about the resistance of MS cements with respect to chloride ingress. Zhang et al. [75] showed that the chloride diffusion coefficient in MS mortars increased with higher w/b ratios and greater sand additions. Nonetheless, the diffusion coefficient of MS mortars was observed to be sixfold lower than that of PC mortars. This was attributed to the significantly smaller porosity and pore size of the MS binder compared to that of the Portland cement, and also confirmed in [20]. Furthermore, only a slight (linear) increase of the migration coefficient with increasing water-to-binder ratio (0.5–0.6) as well as decreasing binder content in the solid part of the mortar (75–25 wt%) was noted for the MS binder systems [75]. No new phases were observed to form and no significant chloride binding on M-S–H, but rather an exclusion of chloride [20].

Studies focusing on PC-based concrete exposed to marine conditions, where M-S–H has formed on the exposed surface, have indicated that the chloride binding capacity of M-S–H (with Mg/Si ratio around 1) is relatively low [76]. Yet, the formation of M-Cl-S–H with significant Cl content has been proposed in a binder with MgCl_2_ and silica fume or siliceous fly ash [77, 78]. However, it is unclear whether the chlorides observed in these works are structural, adsorbed onto M-S–H, or simply due to intermixing with other phases. With respect to phase transformations, a good stability of M-S-H in the presence of chlorides (both NaCl and MgCl_2_) has been suggested [20, 79] at very low (< 0.2 mmol/L) and very high (1.95 wt% NaCl [20]) chloride concentrations. At high (magnesium) chloride contents, the precipitation of magnesium oxychloride phases might occur (MgCl_2_ > 1.5–2 mol∙(kg H_2_O)^−1^) [27, 71], especially at high Mg/Si ratio (> 1.5) [80]. Moreover, the stabilization of MOC phases in diluted systems has been noted in the presence of phosphates [12, 81]. The use of Na-hexametaphosphate (Na-HMP), often used as plasticizer for M-S–H binder systems [82, 83], will introduce phosphate which in turn could stabilize MOC phases at relatively low Cl concentrations.

The presence of Fe and Al, (e.g. originating from the raw materials used, such as Fe from MgO or Al from the silica source), can lead to the formation of Mg–Al/Fe-LDH phases [84]. These phases are known for exchanging anions and may take up chlorides. It has been reported for Mg–Al-LDH [85] that high chloride contents are required for substantial chloride uptake and only limited chloride uptake was reported when carbonate-LDH was present. Furthermore, at lower OH^−^ concentration, as expected for magnesium silicate hydrate and magnesium carbonate binders, chloride uptake was reduced as well [85, 86]. Thus, the impact of chloride uptake by LDH phases in M-S–H or HCB cements is expected to be limited under realistic conditions but must be explored thoroughly in future studies.

### Sulfate attack

Portland cement-based systems can form expansive sulfate products, such as ettringite, in sulfate-rich environments. No such products have been observed in magnesium carbonate binders [18, 87, 88]. For a binder based on MgO with hydromagnesite [18], limited to no additional expansion was observed in 50 g/L Na_2_SO_4_ compared to the expansion due to submersion in H_2_O itself. A slight increase in mass due to sulfate uptake was noted, but its location within the binder phases could not be identified. A slight reduction (< 15%) in strength has been reported for carbonated reactive MgO-based binders exposed to 10% Na_2_SO_4_ [87] and 5% MgSO_4_ [88] solutions. Pu et al. [87] attributed this strength reduction to a slight loss of hydromagnesite, the primary binding phase in their system.

**Fig. 5:**
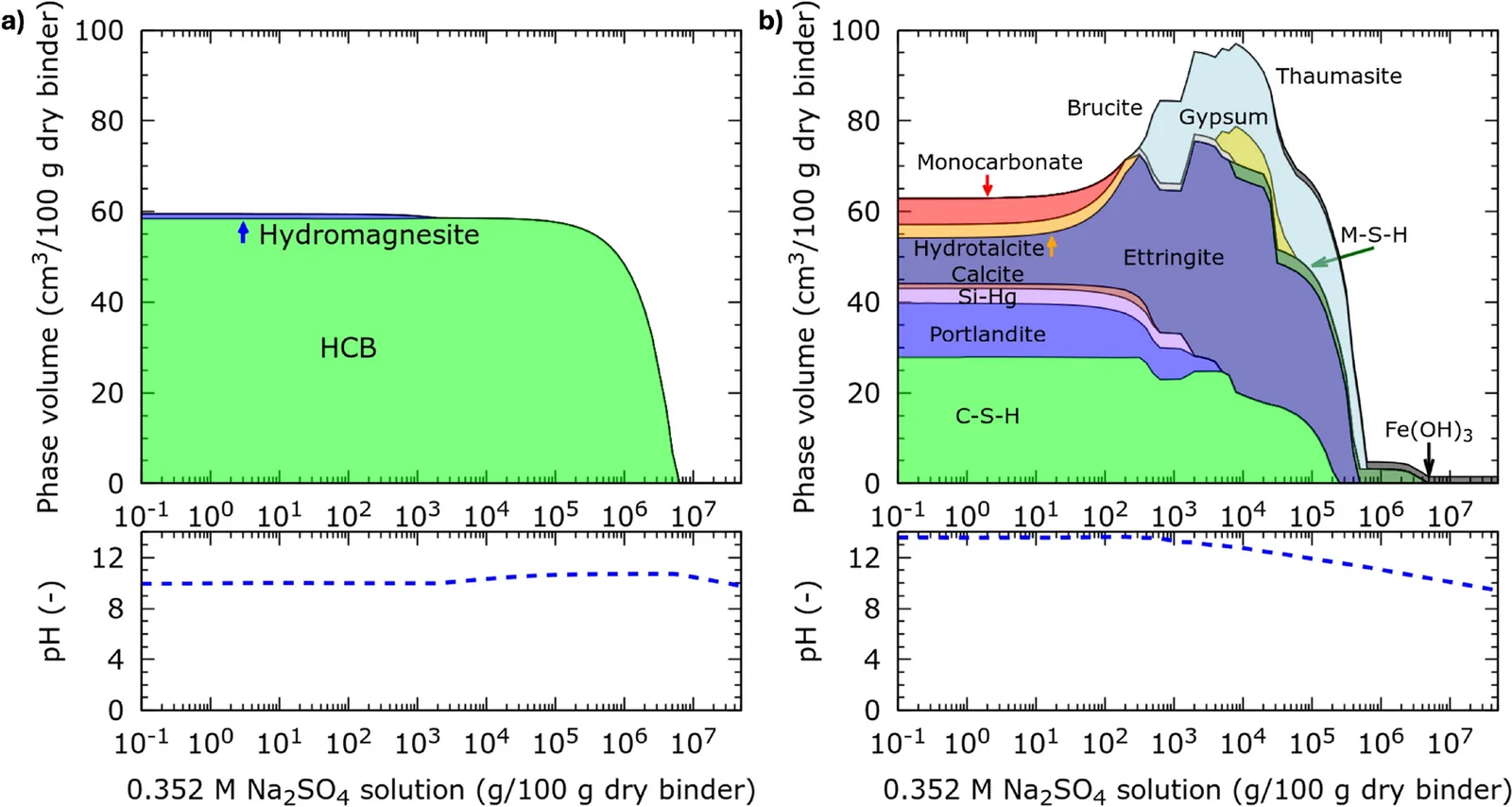
Modelled phase assemblage and associated volume changes upon contact of MgO/hydromagnesite (90/10 mass ratio, water/binder mass ratio = 1) and **b** PC (water/binder mass ratio = 0.4) with 0.352 M Na_2_SO_4_ solution. Data on 3-1-8, 5-1-2 and 5-1-7 MOS phases from [90]. PC modelling using the same modelling strategy as described in [73] using the PC composition from [74]

At the typical concentrations of sulfates used in standard tests (0.35 M Na_2_SO_4_, 50 g Na_2_SO_4_/L), the formation of magnesium oxysulfate phases is not expected. Dinnebier et al. [89] reported the formation of 3Mg(OH)_2_∙MgSO_4_∙8H_2_O (3-1-8) only above 1 mol∙(kg H_2_O)^−1^) sulfate. However, they indicated that this phase is metastable with respect to 5Mg(OH)_2_∙MgSO_4_∙2H_2_O (5-1-2 phase). Li et al. [90] have suggested, based on thermodynamic calculations, that the 5-1-2 phase may be stable at much lower concentrations. Yet, its formation at room temperature at such low concentrations has not been observed experimentally and is thus considered unlikely to form during sulfate exposure in 0.35 M Na_2_SO_4_. This is in agreement with the lack of any MOS phases observed by German [18] after 28 days of exposure of MgO/hydromagnesite binders (mass ratio 90/10 and 70/30) to a solution with 50 g Na_2_SO_4_/L. Thermodynamic modelling of a similar MgO/hydromagnesite (mass ratio 90/10) binder exposed to the same 50 g Na_2_SO_4_/L solution (Fig. 5) also shows that the formation of any MOS phase, even the 5-1-2 phase, is not expected. As in the case of NaCl, leaching is expected at a very high amount of solution. The superior stability of the HCB phase with respect to leaching and sulfate solutions in comparison to hydrated PC is apparent from Fig. 5b.

Also, the effect of sulfate on MS cement and M-S–H is little investigated. Good resistance against sulfate ingress was reported, which was related to the relatively small porosity in M-S–H binders [20]. No new sulfate-containing phases were observed to form and no significant binding on M-S–H, but rather an exclusion of sulfate, similar to the observations reported for chloride [20]. Portland cements exposed to freshwater or seawater can form M-S–H on the leached surface. Detailed investigations of those M-S–H layers indicate no significant binding of sulfate or chloride in M-S–H [76, 91], in agreement with the observation on pure MS cements [20]. As in the case of chloride, M-S–H is also stable in the presence of sulfate (3 wt% Na_2_SO_4_ [20]) without formation of sulfate-containing phases. Only at very high sulfate concentration (MgSO_4_ > 2 mol/kg∙H_2_O) and high Mg/Si ratio [80], the precipitation of magnesium oxysulfates might be expected. In addition, sulfate might be bound in the interlayer of LDH phases [20], if present, as discussed above for chloride.

### Corrosion

The corrosion behaviour of steel reinforcements in MgO-based binders has, thus far, received relatively little attention compared to PC. The pore solution in concrete made from Portland cement is generally alkaline with a pH > 12.5 [92]. This high degree of alkalinity promotes the formation of a thermodynamically stable passive iron (hydr)oxide film on the surface of the steel reinforcement, and results in negligible corrosion rates for carbon steel embedded in PC-based concretes [93]. The pH of pore solutions of MgO-based binders on the other hand is in the circumneutral to mildly alkaline range, pH: 7 to 11 [3] (i.e. significantly closer to the pH region at which steel is thermodynamically unstable). Due to this comparatively low pH of the pore solution, MgO-based cements are often generally *deemed and perceived* to be unsuitable for structural applications in combination with steel reinforcement [3]. However, while the pH is an important criterion to assess the thermodynamic stability of iron, the moisture content at the steel–concrete interface influences the kinetics of iron dissolution due to corrosion [94]. Therefore, factors such as pH, dissolved oxygen, transport and concentration of aggressive species and the moisture content at the steel–concrete interface must be considered complementarily when assessing the corrosion behaviour of the steel reinforcement in MgO-based binders.

The high solubility of the main binding phases in both MOC and MOS can strongly influence the activity of chloride and sulfate anions in solution. Both these anions are known to be severely aggressive towards the steel reinforcement and can lead to significant corrosion [95], at the near neutral pH these binders are stable at [96]. There have been notable cases where MOC cements have been used to produce construction products, and significant corrosion in building elements and fixings occurred due to the presence of Cl^−^ ions [66, 67]. Additionally, in the case of MOC, the high chloride content within the concrete and at the steel–concrete interface can lead to the localised corrosion of the steel. In addition, their low water resistance renders them generally unsuitable for steel-reinforced applications.

**Fig. 6:**
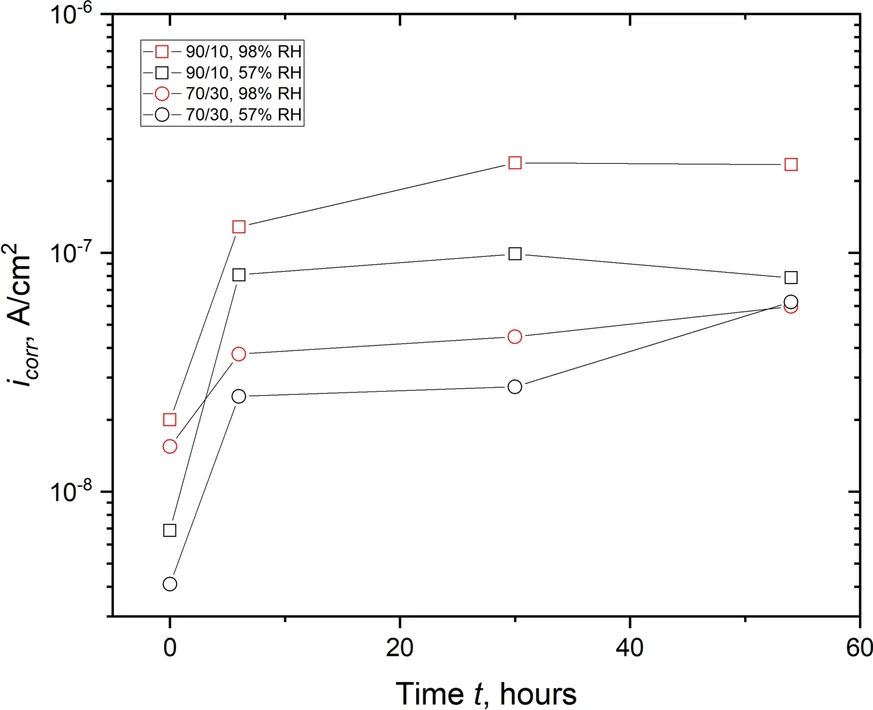
The corrosion rate of carbon steel embedded in MgO-based binders prepared from 90/10 and 70/30 wt% MgO and hydromagnesite, cured at 98 and 57% RH for 28 days, as a function of exposure (partial immersion in water) time. All samples were partially immersed in water after curing (adapted from [68])

Only few studies [53, 97] have investigated the corrosion behaviour of carbon steel in MSH binders. Owing to the low pH of the binder (9.9–10.5), the open circuit potential of the steel reinforcement was found to be much lower in comparison to traditional PC-based concretes. Additionally, when exposed to chloride-containing solutions, the corrosion rate was roughly in orders of tens of μA/cm^2^. Furcas et al. [68] investigated the corrosion of carbon steel embedded in MgO/hydromagnesite mortars exposed to the capillary uptake of water and alkaline, chloride-containing solution across a time span of 3 days. For all sample compositions, exposure and curing conditions investigated, the corrosion current density (*i*_*corr*_) was in the orders of 10^−9^–10^−7^ A/cm^2^ and solely dependent on the moisture state of the mortar (Fig. 6). The corrosion rates are thus comparable to those of passive steel embedded in concretes made from Portland cements, blended cements or other alternative binders [98, 99].

**Fig. 7:**
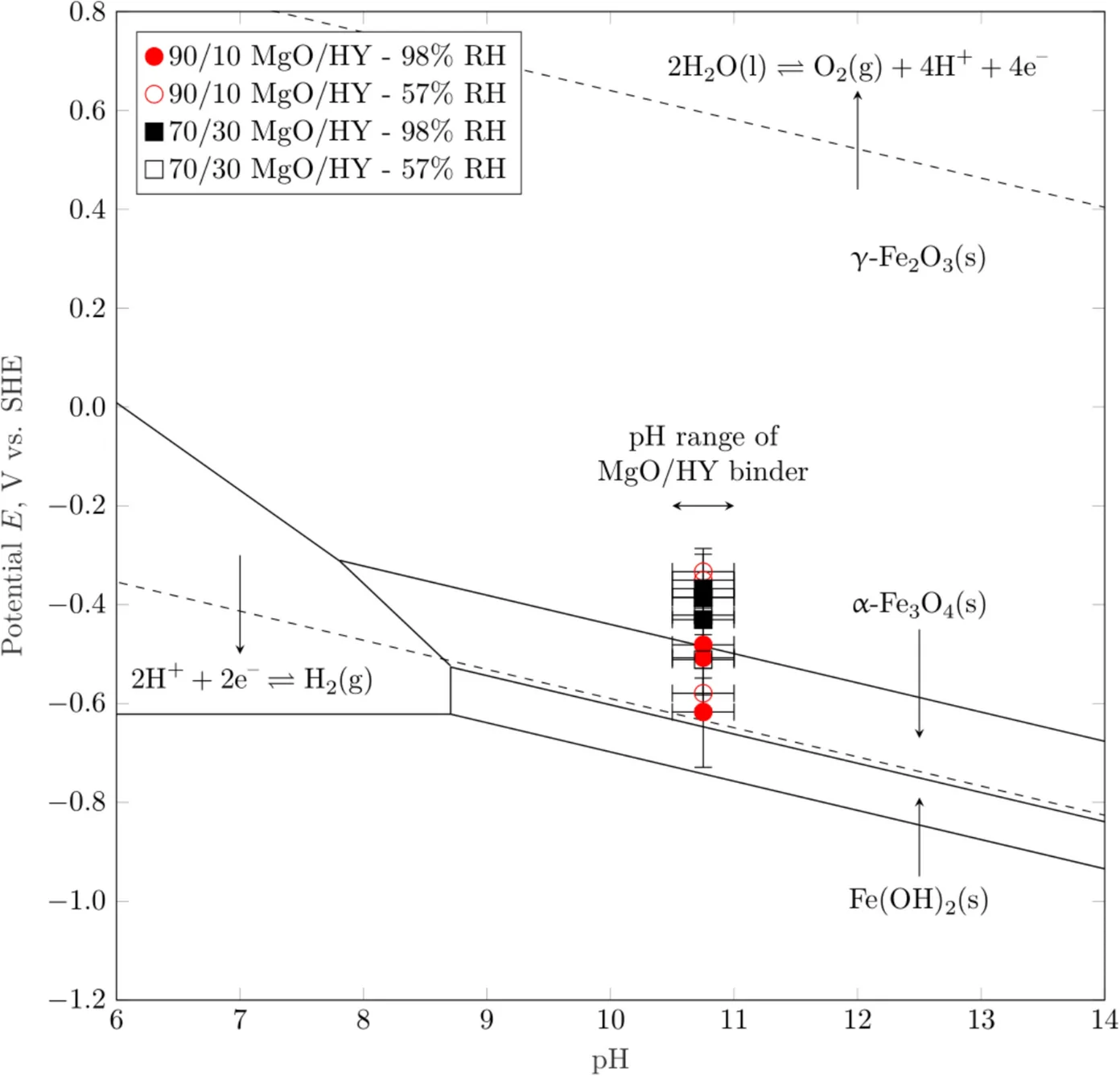
The potential-pH stability diagram of iron at T = 25 °C, together with the open circuit potential measurements of carbon steel embedded in MgO/hydromagnesite mortars. Adapte from Furcas et al. [68]

As further illustrated in Fig. 7, the corresponding open circuit potential measurements of carbon steel embedded in the MgO/hydromagnesite mortar are well within the thermodynamic potential-pH stability regime of various iron (hydr)oxide phases. The obtained corrosion rate and open circuit potential measurements suggest that analogous to the corrosion of steel in carbonated Portland cement concrete [100], the lower pore solution pH of magnesium carbonate concrete is tolerable, as long as the moisture state at the steel–concrete interface can be controlled. However, to assess the suitability of MC cements for reinforcement applications, further long-term corrosion studies are required.

## Outlook and conclusion

The long-term durability of MgO-based cements is considerably less well understood than that of PC-based cements. Significant knowledge gaps remain regarding their long-term durability, particularly in relation to carbonation mechanisms, frost and salt-frost resistance, corrosion of the steel reinforcement, and their interactions with chlorides and sulfates due to the limited numbers of studies carried out. Available evidence in the literature shows that the performance of these binders with respect to different durability indicators is highly dependent on the specific cement chemistry and exposure conditions, with distinct advantages and limitations for each type. Table 1 summaries the key findings for each type of binder with respect to different durability indicators considered in this review. A deeper understanding of different degradation mechanisms, linked to the pore structure, solid phase assemblage, moisture state and pore solution chemistry of MgO-based binders and exposure conditions, is essential to reliably assess their performance against different durability indicators and enable their safe implementation in structural applications from Furcas et al. [68]

**Table 1:**
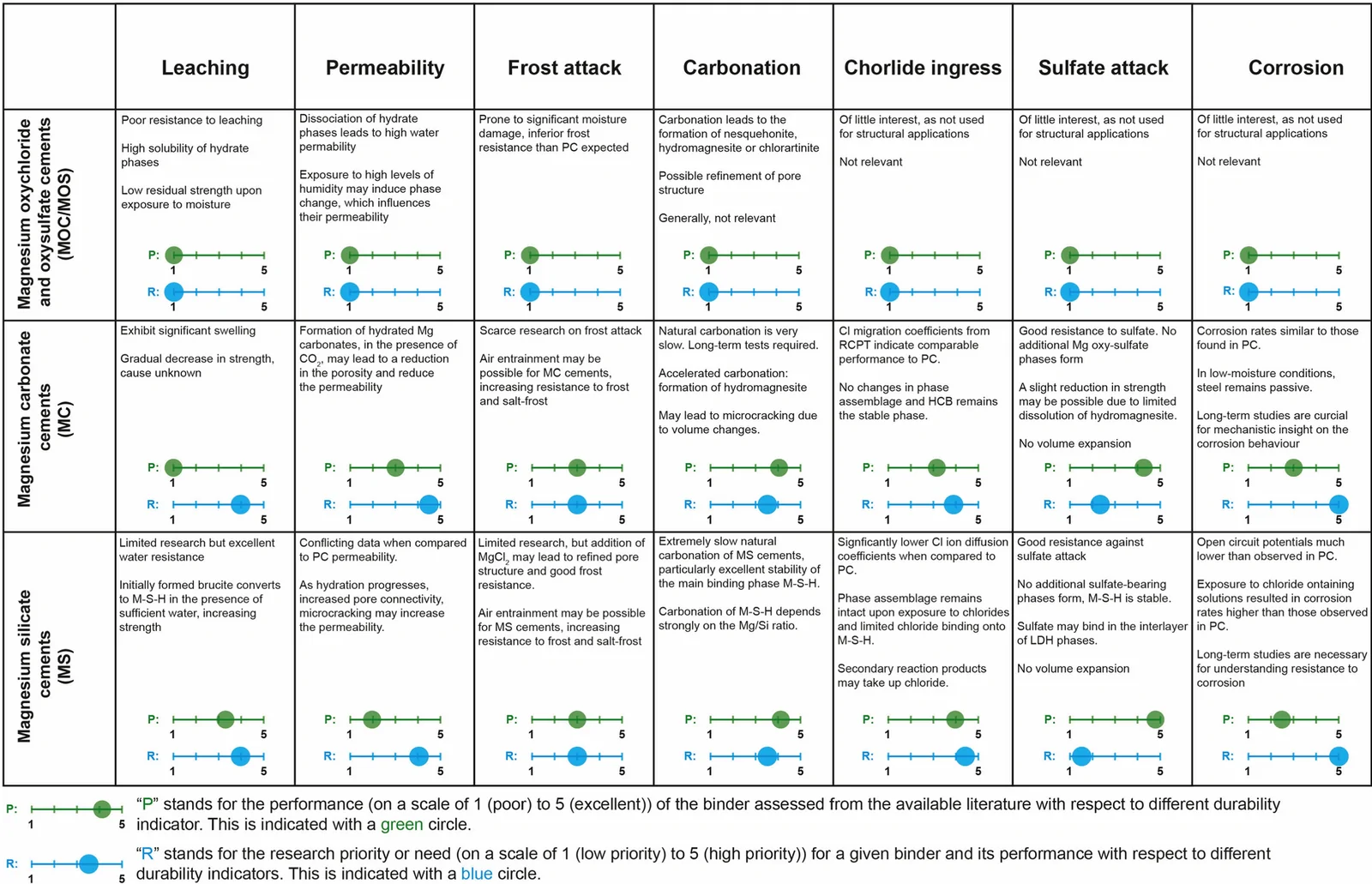
Summary of key durability indicators for magnesium oxychloride/oxysulfate (MOC/MOS), magnesium carbonate (MC) and magnesium silicate (MS) cements

Based on the available evidence in the literature, the performance and research priority/needs of each of the binders with respect to different durability indicators have been highlighted

For MOC and MOS form different metastable and stable hydrous oxysalt phases which are susceptible to leaching under moist conditions as summarized in Table 1. Their sensitivity to moisture and phase instability leads to mechanical instability making them unsuitable for structural applications. The high solubility of the oxysalt phases results in the release of chloride and sulfate anions, posing severe risks of corrosion in moist environments. Although carbonation may refine the pore structure through the formation of magnesium carbonate phases, its net effect on long-term durability is unclear. Consequently, their use has been restricted to niche applications such as wall linings, flooring, and decorative features, where their low density, fire resistance, and high strength can be effectively utilised. Should their use be considered beyond non-structural applications, fundamental studies regarding carbonation, and corrosion in high chloride and sulfate containing pore solutions are essential.

MC cements demonstrate moderate resistance to leaching and permeability, carbonation and chloride ingress occur relatively slowly. Reported swelling and gradual strength loss due to leaching lack mechanistic explanation and require detailed investigation of hydrate evolution and microcracking processes. While carbonation-driven formation of hydrated magnesium carbonates can reduce porosity and permeability, the expansive nature of these phases may compromise structural stability. Frost resistance and salt-frost durability are insufficiently studied (Table 1), despite the potential benefits of air entrainment. Chloride ingress data remain scarce, particularly regarding chloride binding in low-pH pore solutions, while sulfate resistance does not appear to be a major degradation mechanism for these types of cements as no additional sulfate-containing phases have been observed to form. However, long-term validation is essential. Despite their lower pore solution pH in comparison to the pore solutions in PC, electrochemical data reveal low corrosion current densities and passive behaviour of steel, indicating potential suitability for reinforced applications. Long-term studies evaluating the corrosion behaviour are needed for MC cements, and in particular, the role and availability of moisture at the steel–concrete interface needs significant attention.

MS cements show promising intrinsic durability, particularly in terms of water resistance, chloride and sulfate stability; however, available experimental data are limited and sometimes contradictory. Systematic research is needed to clarify permeability evolution during hydration, especially the roles of pore connectivity and microcracking. The resistance of MS cements against frost attack remains a topic that has not received significant attention. Carbonation of MS cements is extremely slow under natural conditions, the carbonation resistance of MS cements strongly depends on the concentration of CO_2_ and the Mg/Si within magnesium-silicate-phases (M-S–H). The Mg/Si ratio plays a critical role in controlling the stability of M-S–H phases, with Mg/Si = ~ 0.7–0.8 showing very high stability upon carbonation. Although chloride diffusion coefficients are generally lower than those of Portland cement, limited chloride binding capacity and low pore solution pH result in elevated corrosion risks for steel reinforcement. Long-term, exposure-based studies linking chemistry, microstructure, transport properties, and corrosion performance are therefore essential for MS cements.

The long-held assumption that a lower pH of the pore solution inevitably leads to depassivation and active steel corrosion, does not fully apply to MgO-based systems. Available studies consistently show that, despite pH values well below those of PC systems, corrosion rates remain relatively low, and steel remains passive. Recent work on steel corrosion in near-neutral to mildly alkaline electrolytes has conclusively shown that other aggressive anions such as Cl^−^ and SO_4_^2−^ in the pore solution, play an important role as well. In addition, electrochemical studies of steel corrosion in MgO-based binders clearly highlight the importance of the moisture state at the steel–concrete interface. These findings underscore that durability assessments of MgO-based binders should also focus on transport processes and exposure conditions, rather than pH alone.

Overall, advancing MgO-based cements toward structural application requires long-term performance data under realistic exposure conditions. Bridging these gaps is essential for assessing the true sustainability and service-life potential of these emerging cementitious systems.
